# Unsupervised Machine Learning Neural Gas Algorithm for Accurate Evaluations of the Hessian Matrix in Molecular Dynamics

**DOI:** 10.1021/acs.jctc.1c00707

**Published:** 2021-10-27

**Authors:** Michele Gandolfi, Michele Ceotto

**Affiliations:** Dipartimento di Chimica, Università degli Studi di Milano, via Golgi 19, 20133 Milano, Italy

## Abstract

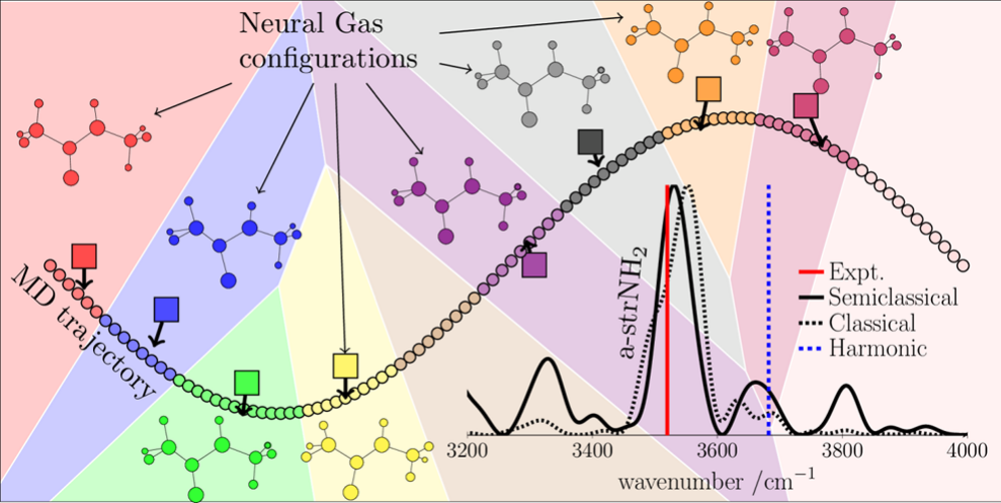

The Hessian matrix of the potential energy of molecular systems is employed not only in geometry optimizations or high-order molecular dynamics integrators but also in many other molecular procedures, such as instantaneous normal mode analysis, force field construction, instanton calculations, and semiclassical initial value representation molecular dynamics, to name a few. Here, we present an algorithm for the calculation of the approximated Hessian in molecular dynamics. The algorithm belongs to the family of unsupervised machine learning methods, and it is based on the neural gas idea, where neurons are molecular configurations whose Hessians are adopted for groups of molecular dynamics configurations with similar geometries. The method is tested on several molecular systems of different dimensionalities both in terms of accuracy and computational time *versus* calculating the Hessian matrix at each time-step, that is, without any approximation, and other Hessian approximation schemes. Finally, the method is applied to the on-the-fly, full-dimensional simulation of a small synthetic peptide (the 46 atom *N*-acetyl-l-phenylalaninyl-l-methionine amide) at the level of DFT-B3LYP-D/6-31G* theory, from which the semiclassical vibrational power spectrum is calculated.

## Introduction

In standard molecular dynamics (MD) simulations, the atomic positions, velocities, and forces are evolved in time according to Hamilton’s equations and calculated at each time-step. The physical interpretation of the atomistic details provided by dynamics simulations is very powerful and finds uncountable applications every day. However, if one looks for a deeper physical insight that requires information about the potential curvature, it becomes necessary to evaluate the Hessian (second-order derivatives of the potential energy) matrix at each time-step. Specifically, Hessians are employed for higher than second-order MD time-integrators,^1−3^ for geometry optimization calculations,^4,5^ for instantaneous normal mode analysis,^6,7^ for accurate force field constructions,^8^ for semiclassical dynamics,^9^ and other applications, such as reaction rate constants with the instanton method.^10,11^ While integration of Hamilton’s equations of motion is doable for any number of degrees of freedom, assuming that the interacting potential is readily available as well as that there is suitable computational power, computing properties that depend on the second or even higher coordinate derivatives of the potential is a challenging task since these calculations usually scale polynomially with the system size. The task may become prohibitive in *ab initio* MD^12^ where the potential and its derivatives are evaluated on-the-fly, that is, by solving the electronic structure problem and using the Hellman–Feynman theorem, or by the finite difference formula using the forces or the potential. To address this issue, a number of approximate methods have been introduced.^13−16^ Usually, these are of the type of updating schemes, where the Hessian is approximated in a step-wise fashion using the latest information available.^17^ These updating schemes were originally developed for optimization^17−21^ (see also references therein) but have much evolved and improved since then. Later, they have been employed in various algorithms for direct dynamics simulations.^1−3^ For example, the Broyden method is based on a first-order Taylor expansion, which is equivalent to the quasi-Newton methods employed in optimization processes. However, in *ab initio* MD, a higher accuracy is desirable as it has been shown how a highly accurate Hessian approximation can attain high simulation quality.^13^ More recently, Denzel and Kästner^22^ followed another route to face the problem, which is to use the Gaussian process regression method^23−25^ to generate a local fit of the potential surface (GPR-PES), possibly using Hessians as fitting variables. Then, the GPR-PES can be differentiated analytically as many times as required, providing accurate Hessian matrices. The method has been successfully employed in various applications ranging from accurate instanton calculations^26^ to the modeling of molecular, amorphous materials and surfaces (see ref (25) and references therein), to mention some. However, the GPR-PES method (including Hessian estimation) was intended to give an accurate description of only a local region of the PES. Hence, it is unsuited for extensive MD simulations. Furthermore, the GPR fitting time and memory usage scale unfavorably with the system size, and the method is not recommended for systems with more than 100 degrees of freedom, as the authors pointed out.^22^

In this paper, we take a different strategy from those described above for the Hessian approximation. The idea is to assign the same Hessian matrix to a group of MD trajectory configurations that are characterized by similar geometric properties. Since the Hessian ultimately depends on the potential energy surface (PES), we think that the collection of molecular coordinates is an appropriate set of variables to combine a group of configurations, given that the Hessian is uniquely defined for each set of atomic coordinates. Specifically, we employ the unsupervised machine learning algorithm “neural gas” to clusterize similar coordinates. The neural gas (NGas) algorithm was originally proposed as a self-organizing-map or a self-organizing-network by Martinetz and Schulten^27^ in 1991, with the objective of learning the dimensions and topology of a generic manifold of simple geometrical shapes and complicated time series.^28^ The algorithm devised by Martinetz, Berkovich, and Schulten features a number of landmark coordinates called neurons that are initialized nearby the objective manifold either randomly or according to some rule. Then, the neurons gradually adapt and connect to best represent the manifold shape, thus arranging in the manifold as an approximate time series. For the adaptation to be effective, the algorithm iteratively drags the neurons closer to the manifold in a way to minimize a given error function, which can be, for example, the sum of the Euclidean distances of each neuron from the collection of events in the time series. As a final result, the manifold is divided into optimal domains, the Voronoi cells, one for each reference neuron.

**Figure 1:**
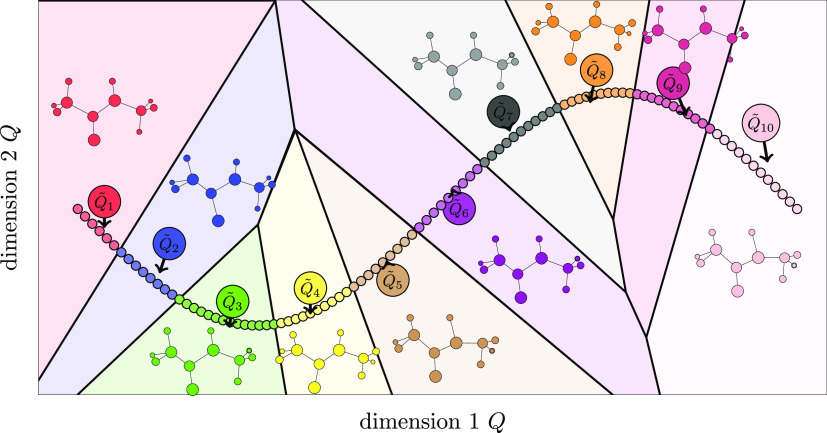
Pictorial representation of neuron adaptation for a MD trajectory manifold in a convenient 2D plane. The trajectory configurations are represented by the collection of small circles, and the neuron positions are the larger circles labeled by *Q̃*_*i*_ for the *i*-th neuron. The arrows at the neuron circles represent the updating coordinate direction. The domains of each neuron are bordered by solid lines and identified by different colors. All geometries within the same domain share the same Hessian matrix, which is the one calculated at the neuron location.

An intermediate step of the algorithm is pictorially represented in Figure 1, where the time series of configurations is reported as a line of small circles and the neuron locations as larger circles. The molecular geometry of each neuron is pictorially represented. The collection of the trajectory configurations that are related to each neuron are distinguished by a color code, and the neuron domains are bordered by continuous black lines. In a few words, molecular geometries of the same color share the same Hessian, which is the one calculated at the corresponding neuron geometry. The time series of configurations which form the manifold can be generated by many trajectories as well. The procedure avoids any redundancy that a multiple trajectory time series may generate when trajectories have crossing paths. This algorithm will take advantage of the fact that bound system trajectories are subject to visit the same phase space neighborhood several times during the dynamics, a point which is missed by the Hessian update schemes (vide infra).^16^ As a matter of fact, neurons undergo a competitive behavior in getting closer to larger portions of manifold conformations, and more probable phase space regions will exhibit higher densities of neurons. Also, we expect that, as shown in Figure 1, for a curved trajectory, the optimal neuron location would be nearby the center of curvature, which is equally representative of the curvature geometries. Instead, when the trajectory lies on a straight line, the optimal neuron location would be on top of the trajectory. A modified version of the NGas algorithm was introduced in 1994 by Fritzke.^29^ In this version, it is not required to specify the number of input neurons, with new neurons being added as the optimization proceeds. Later on, many groups provided further advances and optimizations on top of the original version to enforce topology preservation^30,31^ and allow for a better scaling and optimal growth with increasing amount of data.^31−33^

The use of supervised and unsupervised machine learning algorithms for molecular modeling has a long history in the field of quantitative structure–activity relationships (QSAR).^34^ Different kinds of molecular descriptors^35^ are employed to predict a plethora of properties, especially in the field of medicinal chemistry^36^ and drug discovery.^37,38^ In recent years, supervised algorithms have been recognized as powerful tools in the formal field of theoretical physical chemistry (see ref (39) for an insightful perspective), also for MD simulations.^40−42^ In addition, unsupervised algorithms have found successful applications in (al)chemical space exploration and chemical design.^43−45^

In this paper, we show that the unsupervised machine learning algorithm “neural gas” can optimally compress the information contained in simple molecular geometries along a MD simulation, and we use the compressed information to approximate the Hessian matrix. More specifically, in this work, we develop and test a NGas method for Hessian approximation with applications to the computation of vibrational spectra using the semiclassical initial value representation (SCIVR) method^9,46^ with the divide-and-conquer technique (DC SCIVR) implementation developed by our group.^47^ In fact, the bottleneck of SCIVR dynamics is the computation of the Hessian matrix along the trajectories.

The paper is organized as follows: in the Methods section, we present in detail the NGas method implementation for the Hessian approximation, after recalling other two methods for Hessian approximation that we will compare with. Then we briefly recall the approach we use for the computation of vibrational power spectra in the semiclassical approximation, and eventually in the Results section, we apply the method to several molecular systems of growing dimension up to a small synthetic peptide. We conclude the paper with a summary and discussion of our findings.

## Methods

### Compact Finite Difference Methods

In previous publications,^14,15^ Ceotto, Zhuang, and Hase have presented and showed how to employ a Hessian updating scheme based on a compact finite difference (CFD) strategy for MD simulations.^48−51^ The CFD approach allows one to obtain a high-order finite difference approximation of function differentiations without incurring a large stencil. This goal is achieved by including differentiated terms at more locations within a “compact” stencil. In this updating scheme, the Hessian is estimated by extrapolation. For example, if the MD geometry *X*_*i*_ = (*x*_*i*,1_,*x*_*i*,2_,...,*x*_*i*,*n*_) of *n* scalar entries is followed by *X*_*i*′_ at a later time, the updating scheme *H*(*X*_*i*′_) = *H*(*X*_*i*_) + Δ*H* allows one to estimate the Hessian for the later geometry *H*(*X*_*i*′_) once Δ*H* is estimated. Bofill^21^ proposed the following update recipe
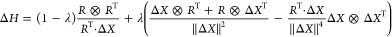
1where λ is a parameter allowed to vary, Δ*X* = *X*_*i*′_ – *X*_*i*_
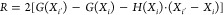
2*G*(*X*) is the gradient and ⊗ and · are the symbols for outer and inner products of vectors.^13^ When λ = 0, the CFD-symmetric rank-one scheme^19^ is derived, while the CFD-power symmetric Broyden scheme is obtained with λ = 1, and the CFD-Bofill family schems is represented by the set of linear combinations between the two. Bofill^21^ suggested the following practical value for λ
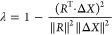
3which avoids the singularity division by near-zero when *R* is almost orthonormal to Δ*X* in the first term of eq 1. This choice was reported to be quite an accurate Hessian approximation.^13^ We provide both our implementation and the pseudocode in the Supporting Information.

### Dynamical Hessian Database Methods

An alternative strategy proposed by our group is to create a dynamical database of Hessians (DBH) and related geometries.^16^ The idea is to approximate *H*(*X*_*i*′_) ≈ *H*(*X*_*i*_) at the MD configuration *X*_*i*′_, whenever *X*_*i*′_ is a geometry close enough to *X*_*i*_, that is, a geometry which has already been saved in a database. Two molecular configurations *X*_*i*_ = (*x*_*i*,1_,*x*_*i*,2_,...,*x*_*i*,*n*_) and *X*_*i*′_ are considered close enough when
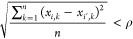
4or
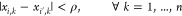
5that is, their distance is smaller than a given threshold ρ. Equation 4 is less strict than eq 5, and hence we adopt the latter (eq 5) for the simulations presented below. To avoid database search latency time, |*x*_*i*,*k*_ – *x*_*i*′,*k*_| can be evaluated mode after mode only for those geometries satisfying the threshold condition. If more than one geometry satisfies eq 5, then the Hessian is approximated by the one associated with the geometry with the smallest difference in eq 5. The database may be updated step by step during the MD simulation, or it may be created once from a given trajectory. In both cases, only the Hessians for those geometries which do not satisfy the requirement in eq 5 are computed, and the corresponding entry is saved in the database. This method has been extensively tested.^16^ The method has allowed for semiclassical simulation of systems where the computational time would have been otherwise too demanding. More details can be found in ref (16). We provide both our implementation and the pseudocode in the Supporting Information of this paper.

### NGas Algorithm for Hessian Approximation

We borrow from the DBH method the idea that close enough molecular configurations have similar Hessians, but we add the feature that the algorithm is allowed to look for optimal configurations even outside the trajectory pathway. As described above, the idea of the NGas method is to approximate a given set of elements with few representative ones, called neurons. In the case of the set of Hessian matrices along a classical trajectory, a NGas algorithm would find few geometries whose Hessians can be employed to approximate the Hessian matrix at every configuration along the trajectory.

Notice that all methods shown in this paper are agnostic with respect to the coordinate system and units. In our case, we usually perform MD either in Cartesian or normal mode coordinates. However, we ultimately employ mass-scaled normal mode coordinates for our spectra calculations. To locate the neurons and proceed with the NGas optimization process, we first scale the whole trajectory set of coordinates to fit a cubic box with edge 1. In other words, we map each mass-scaled normal mode coordinate component *q*_*j*_(*t*) according to the equation
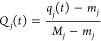
6where *m*_*j*_ = min *q*_*j*_(*t*) is the minimum value in the time series of the *j*-th component, *M*_*j*_ = max *q*_*j*_(*t*), and the new coordinates **Q**(*t*) are the scaled coordinates with values between 0 and 1. Once the number of neurons, that is, the number of most representative geometries, is chosen, we evenly sample the initial guess *s* for the neuron positions directly from the set **Q**(*t*) of the trajectory geometries, that is, initially the set {**Q̃**} ⊂ {**Q**(t)}. In practice, a fixed number of neurons are initialized on top of the trajectory configurations in normal mode coordinates and distributed at fixed time intervals. In their first presentation of the NGas algorithm, Martinez and Schulten^27^ suggested that at each epoch τ, all trajectory geometries **Q**_*i*_ are sampled in a random order from the set of trajectory configurations {**Q**(*t*)} (without repetition). Every time a configuration **Q**_*i*_ is sampled, each *j*-th neuron **Q̃**_*j*_ is updated according to following rule
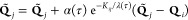
7where *K*_*ij*_ is an integer number that ranks the distance between the trajectory scaled coordinate geometry **Q**_*i*_ and the neuron **Q̃**_*j*_. Specifically, *K*_*ij*_ is equal to 0 for the nearest trajectory geometry and to (*n* – 1) for the furthest one. In eq 7, α(τ) and λ(τ) are parameters which are modeled to decrease during the optimization process. These parameters change for each epochal iteration, and they tune the NGas adaptability, that is, its ability to expand and how fast this expansion is performed. More specifically, λ tunes the number of neighbor coordinates that can significantly interact with each neuron, while α tunes the adaptability of the NGas. In other words, the larger the λ, the greater the number of trajectory geometries that significantly contribute to the updating scheme in eq 7, while α tunes how large the response of **Q̃** is and after how many iterations it is still responsive and learning. α and λ are updated at each epoch with the same rule^28^
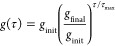
8with *g*_init_ and *g*_final_ being parameters. Reasonable choices for these parameters are α_init_ = 0.3, α_final_ = 0.05, λ_init_ = 30, and λ_final_ = 0.01, independently of the simulated system.^28^

The updating formula in eq 7 can also be written using the  operator formalism that we introduce here
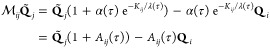
9where α(τ) and λ(τ) have been grouped into one parameter *A*_*ij*_(τ) = α(τ) e^–*K*_*ij*_/λ(τ)^, which depends on α, λ, and *K*_*ij*_. *A*_*ij*_(τ) has the form of a Boltzmann factor with temperature λ(τ), and it is interpreted as a kind of neuron “influence probability.” As the NGas training goes on, λ(τ) (the analogous of temperature) decreases and the gas freezes nearby the trajectory. Assuming that we know beforehand all *K*_*ij*_ coefficients for the motion of the neuron **Q̃**_*j*_ (in general we do not), by applying the  operator of eq 9 for *N*_steps_ time-steps, that is, for the whole set of trajectory points {**Q**(*t*)}, in a random order and without repetition, one gets
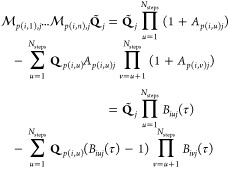
10where *p*(*i*,*u*) is the *u*-th element of a random permutation of the index *i* over the list of the first *N*_steps_ natural numbers, and we wrote the matrix with permuted indices as a three indice tensor: *B*_*iuj*_(τ) = 1 + *A*_*p*(*i*,*u*)*j*_(τ). Equation 10 accounts for the core functionality of the NGas algorithm. In eq 10, the first term on the right-hand side does not depend on the trajectory position but only on the trajectory distance ranking from the *j*-th neuron **Q̃**_*j*_, in the form of the *K*_*ij*_ coefficients. Instead, the second term in eq 10 depends on the trajectory position **Q**_*p*(*i*,*u*)_ both explicitly and implicitly through *K*_*ij*_. Eventually, when λ tends to 0 as described above, also *A*_*ij*_ goes to 0 and the sums and products in eq 10 converges to a final neuron position with less than *N*_steps_ terms. Within the analogy of the NGas, we would say that in the case when the gas is cold, it is influenced only by the local manifold (nearby trajectory points) rather than by the whole environment (the entire trajectory points), even if all trajectory configurations are summed by the index *u* in eq 10. However, even if eq 10 has been introduced to better understand the physics of the NGas iterations, eq 7 is employed in practice. According to these equations, the NGas process is a competitive type of learning since neurons compete to be nearest as possible to the trajectory geometries. This competitiveness is encoded in the parameters *K*_*ij*_, which may change every time a neuron is moved and make it impossible to use eq 10 straightforwardly.

Once the gas is frost, we perform further optimization of each neuron position **Q̃**_*j*_. First, we consider that for each trajectory point **Q**_*i*_, there is only one nearest neuron position **Q̃**_*j*_. Then, we collect all these points into a set {**Q**(*t*)}_*j*_ which is the collection of trajectory segments nearest to the *j*-th neuron, and there will be as many sets of this type as the number of neurons. Eventually, we can estimate the error *E*(**Q̃**_*j*_) to consider the neuron **Q̃**_*j*_ at the place of the trajectory segments **Q**(*t*) as the line integral
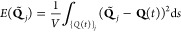
11where *V* = ∫_{*Q*(*t*)}_*j*__d*s* is a normalization constant and d*s* is the integration line segment. Now, we can locate **Q̃**_*j*_ such that *E*(**Q̃**_*j*_) is minimal. The first-order derivative of E(***Q̃***_*j*_) with respect to each **Q̃**_*j*_ is
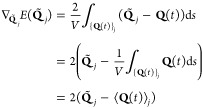
12

Equation 12 is equal to 0 when **Q̃**_*j*_ is equal to the “center of mass” ⟨**Q**(*t*)⟩_*j*_ of the trajectory segments {**Q**(*t*)}_*j*_. Hence, we implemented into the algorithm this further optimization step such that each neuron is eventually placed at the center of mass with respect to the trajectory points associated with that neuron.

**Figure 2:**
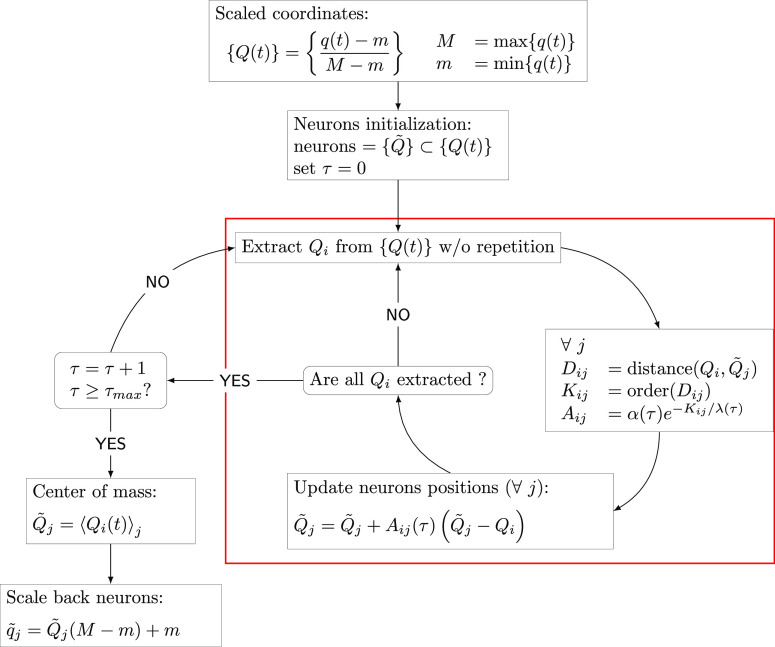
Flow diagram of our NGas implementation. Neurons are sampled from the scaled coordinates and iteratively optimized according to the cyclic part of the diagram. Every time a coordinate **Q**_*i*_ is sampled, one needs to compute its distance from every neuron to determine the ordering (encoded in the **K** matrix). Once the training is done, the neurons are scaled back to their original normal mode or Cartesian form. A red rectangular frame delimits the core part of the NGas algorithm, where neurons are updated in competition with one another to get closer to the trajectory.

Figure 2 reports the flow diagram of the algorithm described above, with the core part of the algorithm enclosed by the red rectangular frame. Apart from the scaling and the final optimization steps, the algorithm can be traced back to the first version by Martinetz and Schulten.^27^ At each epoch, all trajectory coordinates **Q**_*i*_ enter in a random order the NGas optimization cycle, where the distance *D*_*ij*_ from its nearest neuron **Q̃**_*j*_ is evaluated together with the order coefficient *K*_*ij*_. The epoch step is completed only after all trajectory points have been considered and the related neuron updated according to eq 7. For the following epoch, *A*_*ij*_(τ) is updated and so on. At the end of the epoch evolution, each *j*-th neuron coordinate is placed at the center of mass of the collection of trajectory points that are nearest to that neuron {**Q**(*t*)}_*j*_. The new location **Q̃**_*j*_ is then transformed back into the original trajectory coordinate system of reference, **q̃**_*j*_, that can be either Cartesian or normal mode ones. Recently, an algorithm^52^ that uses the idea of dividing the configuration space in Voronoi cells (as in the NGas method) has been proposed. The algorithm creates an *on-the-fly* updated mesh to approximate the potential energy from previous potential and potential gradient evaluations.

We evaluate the quality of the approximation as the mean absolute error (MAE) of the Cartesian Hessian matrix elements
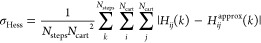
13where *N*_cart_ is the number of Cartesian coordinates, *N*_steps_ is the number of MD time-steps, *H*_*ij*_(*k*) is the entry of the exact Hessian matrix, and *H*_*ij*_^approx^(*k*) is the approximated one, both at step *k*. We provide both our implementation and the pseudocode in the Supporting Information.

## Semiclassical Initial Value Representation Vibrational Spectroscopy

In this paper, we will employ the NGas approximation described above for the calculation of Hessians in semiclassical dynamics for spectroscopy calculations. The semiclassical power spectrum *I*(*E*) of a system of Hamiltonian *Ĥ* can be written as the Fourier-transformed wavepacket survival amplitude (in atomic units)^53−55^
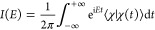
14where |χ(*t*)⟩ = e^–i*Ĥt*^|χ⟩ is the quantum time-evolution of the arbitrary reference state |χ⟩. The power spectrum provides the collections of all vibrational eigenvalues on an absolute scale. We calculate eq 14 using the time-averaging semiclassical initial value representation (TA SCIVR) method,^9,56−64^ where a time-averaging filter is applied to the semiclassical Heller–Herman–Kluk–Kay (HHKK) propagator.^65−77^ The TA SCIVR expression of eq 14 for a system characterized by *N*_vib_ degrees of freedom is
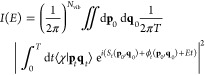
15where *T* is the total simulation time, *S*_*t*_(***p***_0_,***q***_0_) is the instantaneous action of the classically evolved trajectory (**p**_*t*_,**q**_*t*_), and the phase-space integration is performed on the initial trajectory momenta ***p***_0_ and positions ***q***_0_. In eq 15, |***p***_*t*_***q***_*t*_⟩ are coherent states with the following expression in position representation^78^
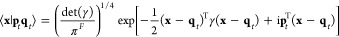
16where **γ** is an *N*_vib_ × *N*_vib_ diagonal matrix, whose elements are equal to the harmonic frequencies of the system. In eq 15, ϕ_*t*_(***p***_0_,***q***_0_) is the phase of the HHKK prefactor^46,79^
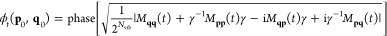
17where ***M***_***ij***_, with ***i****,****j*** = ***p***, ***q***, are the elements of the symplectic (monodromy or stability) 2*N*_vib_ × 2*N*_vib_ matrix
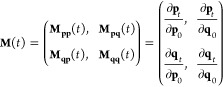
18

Following Hamilton’s equations, the time-evolution of **M**(*t*) is
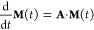
19where
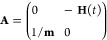
20and where **H**(*t*) is the Hessian matrix at time *t*. Thus, it is necessary for an accurate Hessian approximation to have an accurate **M**(*t*) matrix and an accurate vibrational power spectrum. We monitored the accuracy of **M**(*t*) by exploiting its symplectic property and checking the deviation of its determinant (or better, the determinant of the positive-definite matrix **M**^T^(*t*)**M**(*t*)) from unity.

To beat the curse of dimensionality, we reduced the phase space integration of eq 15 to a few, or just one, trajectory simulations, where each trajectory starts from the global minimum and with an energy equal to the harmonic vibrational energy level that one is looking for.^80^ This method is called multiple coherent SCIVR (MC SCIVR),^81,82^ and it exploits the fact that during the simulation, the delocalization of the coherent states will account for anharmonicity and reproduce the anharmonic vibrational peak position.^14,15,83−95^ The method also allows to identify each mode contribution by selecting a suitable combination of coherent states obtained by changing the sign of the momentum part of the coherent state.^96^ This method has been recently further improved by introducing the DC SCIVR method. The DC basic idea is to calculate the full-dimensional spectrum as the composition of subdimensional ones using eq 15 but with reduced dimensionality phase space quantities. In eq 15, the potential, which is a part of the action, is the only quantity that cannot be exactly projected in a reduced dimensionality space. For this reason, we have introduced the following approximation for the partial *M*-dimensional spectrum
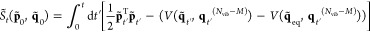
21where the tilde ∼ indicated a *M*-dimension quantity, V(**q̃**_*t*′_,**q**_*t*′_^(*N*_vib_–*M*)^) is the full dimensional potential, and V(**q̃**_eq_,**q**_*t*′_^(*N*_vib_–*M*)^) is the one obtained by fixing at equilibrium the coordinates in the *M*-dimensional subspace. This approach has been successfully applied to several high-dimensional complex systems, including fluxional ones, like small water clusters^97^ and the protonated water dimer.^98^ When combined with MC SCIVR by projecting few full-dimensional classical trajectories into sub-dimensional phase space components, we obtain the MC-DC SCIVR, which can deal with very high-dimensional systems. Notable applications of MC-DC SCIVR include dipeptide derivatives,^99^ nucleobases^100^ and nucleosides,^101^ water clusters up to (H_2_O)_23_,^102,103^ and molecules adsorbed on titania surfaces.^104^ In the DC SCIVR method, one needs to properly partition the full-dimensional vibrational space. One can reach this goal by coarse-graining the time-averaged Hessian matrix or by splitting the Jacobian (monodromy) matrix **M**(*t*) in square blocks, such that the determinant of each block is as close as possible to 1, in partial satisfaction of Liouville’s theorem. In either case, the result is a block diagonalized matrix, where each block represents a vibrational subspace. If one chooses to use the Jacobian approach, the probability graph–evolutionary algorithm (PG–EA) that we recently reported is the way to go.^105^ The PG–EA algorithm uses a cluster graph representation of the molecule’s normal modes, where connected modes are within the same subspace. Such a representation is particularly advantageous because it is invariant with respect to the permutation of modes and subspaces.

## Results

In this section, we present simulations of growing complexity, starting from the small molecular systems H_2_O, HCOH, and CH_4_, going to the smallest prototype of peptide bond [*trans N*-methylacetamide (NMA)], up to a small synthetic peptide (*N*-acetyl-l-phenylalaninyl-l-methionine amide), which is composed of 46 atoms and 132 vibrational degrees of freedom. All simulations consist of a single 3000 time-step constant energy (*NVE*) classical trajectory with a 10 a.u. constant time-step. The initial conditions are chosen according to the MC-DC SCIVR recipe described above. The classical equations of motion are integrated using a four-order symplectic integrator.^106^ We employed precomputed PESs for H_2_O,^107^ HCOH,^108^ CH_4_,^109^ and NMA,^110^ while the *N*-acetyl-l-phenylalaninyl-l-methionine amide (Ac-Phe-Met-NH_2_) molecule was simulated on-the-fly by direct *ab initio* MD at the level of DFT-B3LYP-D/6-31G* theory. The PES derivatives are computed by finite differences with a fixed displacement of 10^–3^ a.u. In the case of the *trans* NMA calculation, we employ an analytical gradient PES,^110,111^ and the Hessian matrix is computed by the finite difference of the gradient. The NGas method has been optimized using 150 neurons for the simulation of H_2_O, HCOH, CH_4_, and NMA, and 300 neurons are employed in the case of Ac-Phe-Met-NH_2_. The number of learning epochs and the α_init_, α_final_, λ_init_, λ_final_ parameters are kept fixed, as described in the Methods section. We performed all computations reported here on a computer laptop using a single core [Intel(R) Core(TM) i7-4510U CPU@2.00GHz, with less than 16 GB of available memory] with the exception of Ac-Phe-Met-NH_2_, whose Hessians have been computed on the group computer cluster using 10 cores [Intel(R) Xeon(R) CPU E5-2660 v3@2.60GHz 125Gb] per Hessian matrix.

### Hessian Approximation Accuracy

To test the accuracy of each method, we performed calculations where 150 Hessians (300 in the case of Ac-Phe-Met-NH_2_) out of 3000 time-steps (2500 in the case of Ac-Phe-Met-NH_2_) are calculated explicitly, that is, from the PES or the electronic structure, and the remaining ones are approximated. In others words, exact Hessians are estimated every 20 (about 8 in the case of Ac-Phe-Met-NH_2_) MD time-steps, and all others are approximated. The deviations of the approximated Hessians from the exact ones are estimated using eq 13. Notice that, although the Hessians in eq 13 are in Cartesian coordinates, we employ normal mode coordinates in the DBH and NGas methods to locate the optimal configurations.

Table 1 reports the results of this test for each molecule, and it shows that the computational time of the Hessian matrix calculation is the simulation bottleneck when evaluated by *ab initio* methods. Actually, when using a precomputed PES, the time required for the NGas algorithm iterations is roughly of the same order of magnitude of evaluating the Hessian for each trajectory configuration. To appreciate the advantage of the approximation schemes in terms of cpu-time, one has to reach the 30 degrees of freedom of the NMA molecule. However, even in this case, the use of analytical gradients provided by the precomputed PES^110^ accelerates the Hessian matrix estimation and keeps the option to evaluate all Hessians along the trajectory viable. We can see a clear advantage of the approximation methods only when dealing with Ac-Phe-Met-NH_2_, which we simulated on-the-fly. In this case, the evaluation of a single Hessian (SH) matrix takes about 3 h with NWChem package^112^ on a 10 core [Intel(R) Xeon(R) CPU E5-2660 v3 @ 2.60GHz 125Gb] node, and the NGas and DBH methods become, in this case, the only viable option. Table 1 reports also in the fourth column the error σ_Hess_ for each method with respect to the all-Hessian evaluation. We notice that the NGas method is as accurate as the DBH one in the case of Ac-Phe-Met-NH_2_. In the fifth column of Table 1, the relative σ_Hess_ shows how each method compares with the NGas in terms of accuracy. We see that by using the NGas method, we can decrease the error by about 26% for small molecular systems, while the NGas error is comparable with the DBH method in the cases of the NMA and Ac-Phe-Met-NH_2_ molecules. We can understand this trend, considering that the higher the number of degrees of freedom, the less often the trajectory visits the same phase space region. In these cases, the NGas method provides a solution that is very similar to the DBH one since neurons are distributed along the trajectory and basically coincide with the molecular geometries at which Hessian matrices are calculated according to the DBH approach.

**Table 1:** Accuracy and Computational Time for Different Hessian Approximation Methods^a^

molecule	#Hessians	method	10^2^σ_Hess_	relative σ_Hess_^b^	method cpu-time	Hessians cpu-time	total cpu-time
H_2_O	150	NGas	0.539	1.00	19.314	0.197	19.51
	150	DBH (ρ = 2.59)	0.728	1.35	0.355		0.55
	150	Bofill	2.336	4.33	0.148		0.346
	3000	all Hessians	0.000	NA	0.000	3.947	3.95
HCOH	150	NGas	0.612	1.00	19.395	0.356	19.75
	150	DBH (ρ = 8.22)	0.824	1.34	0.329		0.69
	150	Bofill	1.570	2.56	0.160		0.52
	3000	all Hessians	0.000	NA	0.000	7.115	7.12
CH_4_	150	NGas	0.732	1.00	18.923	0.492	19.42
	150	DBH (ρ = 7.95)	1.000	1.37	0.345		0.84
	150	Bofill	2.231	3.05	0.192		0.68
	3000	all Hessians	0.000	NA	0.000	9.835	9.84
NMA	150	NGas	0.447	1.00	22.582	12.842	35.42
	150	DBH (ρ = 21.15)	0.490	1.09	0.499		13.34
	150	Bofill	0.935	2.09	0.262		13.10
	3000	all Hessians	0.000	NA	0.000	256.834	256.83
Ac-Phe-Met-NH_2_	298	NGas	0.059	1.00	27.667	7620.819^c^	7621.28^c^
	298	DBH (ρ = 11.9)	0.059	1.00	2.697		7620.86^c^
	312	Bofill	0.153	2.58	2.030	7978.845^c^	7978.88^c^
	2500	all Hessians	0.000	NA	0.000	63,933.049^c^	63,933.05^c^

a First column is the molecule, the second column is the number of exact Hessian calculations, the third column is the Hessian approximation method, the fourth column is the error according to eq 13, the fifth column is the relative error respect to the NGas method, the sixth column is the cpu-time for each method, the seventh column is the cpu-time for the exact Hessian evaluation, and the last column is the total computational time. All times are in seconds, except explicitly indicated. For each molecule, the NGas, the DBH at threshold ρ, and the CFD (Bofill) methods are tested. The “all Hessians” label is for Hessian calculations at each time-step, that is, without any approximation.

b Defined as the error of the method divided by the error of the NGas method.

c Core hours (average of core hours necessary for the computation).

Overall, we can observe that the ratio of computational time *versus* the number of degrees of freedom is almost constant for all methods, and it increases moderately only in the case of the Ac-Phe-Met-NH_2_ system. This is mainly due to the time required to store and copy the trajectory. At this stage, we cannot assert what will happen for even higher-dimensional systems. However, we still can test the robustness and stability of each method by decreasing the number of PES or *ab initio* Hessian entries. In this way, we can also better understand which are the minimum number of Hessian evaluations necessary for obtaining an accurate estimate. We focus on the Ac-Phe-Met-NH_2_ system and on the NGas and DBH approximations. Table 2 reports the values of σ_Hess_ of eq 13 for the two methods for the different exact Hessian evaluation times reported in the second column. Clearly, the more the *ab initio* Hessians are computed, the smaller the approximation error is, as reported in the third column. If the NGas and DBH errors are compared for about 200 exact Hessian evaluations, DBH is more and more accurate as the number is significantly reduced down to 25. We think that this poor performance of the NGas method is due to the fact that, given the extremely low numbers of Hessians provided, the neuron locations are not representatives of their trajectory neighborhood. In other words, when the system conformation is averaged over many ones, the result may be very different from the actual conformations visited along the classical trajectory. To improve and going beyond this limitation, we use an extended set of variables for the neurons’ space, which includes also the potential gradients in the NGas training process. While the original neurons are identified by a set of normal mode molecular coordinates of the type **q̃** = (*q̃*_1_,...,*q̃*_*N*_vib__) in the improved version, the vector which identifies the neuron includes the energy gradient coordinates as well, . This extended neuron set of variables accounts for the PES slope in addition to the molecular positions. In this way, unrealistic conformations with huge internuclear forces (and consequently large Hessian elements) are excluded in favor of more realistic conformations. The improved results are reported in the last column of Table 2. The extended NGas is always more accurate not only with respect to the original NGas method but also to the DBH method, in particular, for the cases when there are few exact Hessian estimates. We observe again that when the number of neurons is increased, these are allowed to have a neighboring trajectory segment that is a straight line, and thus the NGas and DBH methods become alike.

**Table 2:** Hessian Element MAE from Eq 13 (σ_Hess_) for the NGas and DBH Methods by Varying the Number of Exact Hessian Evaluations Indicated in the Second Column in the Case of the Ac-Phe-Met-NH_2_ Molecule

		10^2^σ_Hess_		relative σ_Hess_^b^
method	#Hessians	*q̃*^a^	q̃ ∪ ∇q̃^a^	q̃ ∪ ∇q̃^a^
NGas	25	0.372	0.260	1.00
DBH (ρ = 55.0)	25	0.319		1.23
NGas	50	0.357	0.219	1.00
DBH (ρ = 40.0)	50	0.267		1.21
NGas	100	0.157	0.153	1.00
DBH (ρ = 27.3)	100	0.167		1.09
NGas	200	0.090	0.089	1.00
DBH (ρ = 17.3)	200	0.090		1.01

a The columns “*q̃*” and “*q̃* ∪ ∇*q̃*” refer to different NGas training spaces, as described in the text, while the last column reports the relative error with respect to the best NGas estimate.

b Defined as the error of the method divided by the error of the NGas method.

Although the NGas method seems to provide a small improvement with respect to the DBH one for the larger systems, that is, NMA and Ac-Phe-Met-NH_2_, we can prove that it can reach an accuracy comparable to that one observed for the smaller systems. In fact, both NGas and DBH methods approximate only the regions of configurational space that are close to the trajectory since they are based on the distance from neighboring geometries. Hence, if we employ 150 neurons to approximate a 3000 step trajectory, each Voronoi cell contains on average 20 geometries and the related Hessians. When the system becomes larger, we expect these geometries to be visited within the same portion of the trajectory. This is the reason why DBH and NGas methods provide more and more similar results as the system size grows. However, things are different if we use an ensemble of MD trajectories because in this case, it is very likely that trajectories cross and overlap significantly, as in a tangle of strings. Table 3 reports the numerical results of two ensembles of trajectories.

**Table 3:** Hessian Element MAE of Eq 13 (σ_Hess_) for the NMA Molecule Using the NGas and DBH Methods Obtained by Varying Either the Total Number of configurations (second Column) or the Number of *ab initio* Hessians (Third Column)

method	configurations (#trajectories × #steps)	#Hessians	10^2^σ_Hess_	relative σ_Hess_^a^
NGas	100 × 1000	999	1.33	1.00
DBH (ρ = 54.7)	100 × 1000	1008	1.64	1.23
NGas	500 × 1000	1000	1.52	1.00
DBH (ρ = 67.6)	500 × 1000	999	1.94	1.28
NGas	100 × 1000	999	1.33	1.00
DBH (ρ = 45.6)	100 × 1000	2049	1.34	1.01
NGas	500 × 1000	1000	1.52	1.00
DBH (ρ = 47.5)	500 × 1000	6082	1.56	1.03

a Defined as the error of the method divided by the error of the NGas method

The trajectory initial conditions were sampled from the Husimi distribution in phase space centered at the equilibrium values. We notice that the trajectories originated by this distribution are spread in energy values, in contrast to previous simulations, and the errors in the Hessian matrix are inevitably higher. In the upper part of Table 1, the NGas method provides a smaller value of σ_Hess_ for the same number of *ab initio* Hessians employed in the DBH simulation. In the lower part of the table, the same average error in the Hessian matrix is reached only when DBH employs more than 6 times *ab initio* Hessians than the NGas method. With about 500 thousand geometries and one thousand neurons, our implementation of the NGas method takes its toll, and the training of the NGas takes about 7 h to be optimized compared to the 50 min required by DBH. However, if one takes into account the ∼10 min per core [Intel(R) Xeon(R) CPU E5-2660 v3@2.60GHz 125Gb] that it takes to compute the *ab initio* Hessian matrix of NMA at DFT-B3LYP/6-31G* level of theory, it is still convenient to use the NGas method. Finally, the σ_Hess_ values reported in Table 3 are rather large as we enforced less than one *ab initio* Hessian matrix every 100 Hessians, which is quite a drastic setup.

### Spectroscopic Simulations

One may wonder which level of Hessian approximation accuracy is requested in MD applications and how important the choice of the approximation method is. To reply to this question, we decided to employ our approximate Hessians for the integration of eq 19 and the calculation of the power spectrum using eq 15. Specifically, we simulated the full-dimensional vibrational power spectrum of the small Ac-Phe-Met-NH_2_ peptide using a single on-the-fly *ab initio* trajectory with our MC-DC SCIVR method, described in the Semiclassical Initial Value Representation Vibrational Spectroscopy section. In the DC strategy, we need to find a vibrational space subdivision, which is the result of a trade-off between spectroscopic accuracy and feasibility. Too high-dimensional vibrational subspaces are not practical, but too low-dimensional ones may turn out to be a drastic approximation. For these reasons, we performed a preliminary coarse-graining of the time-averaged Hessian matrix by fixing to zero all elements smaller than 8.0 × 10^–6^ a.u.^113^ In this way, after conveniently permuting rows and columns, we obtained a block diagonal matrix whose 23-dimensional subspace contains all stretching modes of the amine group we are interested in. These are denominated as sNH_2_ (mode number 129), NH(II) (130), NH(I) (131), and aNH_2_ (132). We focus on these fundamentals because their experimental values are available for comparison.^114^ This subspace is further decomposed into smaller subspaces using our PG–EA algorithm.^105^ The stretches we are interested in are highlighted in bold in the normal mode subspaces {10 30 33 36 37 38 42 46 **130 131**} and {47 105 **129 132**}. The mode numbers are assigned according to the harmonic frequency values, where smaller numbers mean lower harmonic frequency values. Both subspaces contain floppy modes. In particular, the first subspace contains several floppy modes, and we expect that the partial spectra of the NH(II) (130) and NH(I) (131) modes will embody several combination features of these stretches with floppy modes.

**Figure 3:**
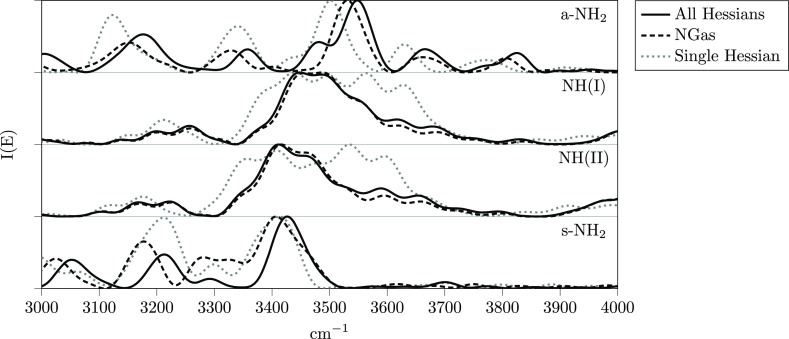
Spectroscopic Hessian accuracy test for the NH_2_ stretches in the amide group of the Ac-Phe-Met-NH_2_ peptide. The dotted line is for the SH approximation;^115^ the dashed line is for the NGas approximation including the gradient information with 200 neurons and 200 *ab initio* Hessian calculations. The continuous line, which is labeled as “all Hessians,” reports the simulation where all Hessians are obtained from *ab initio* calculations.

**Figure 4:**
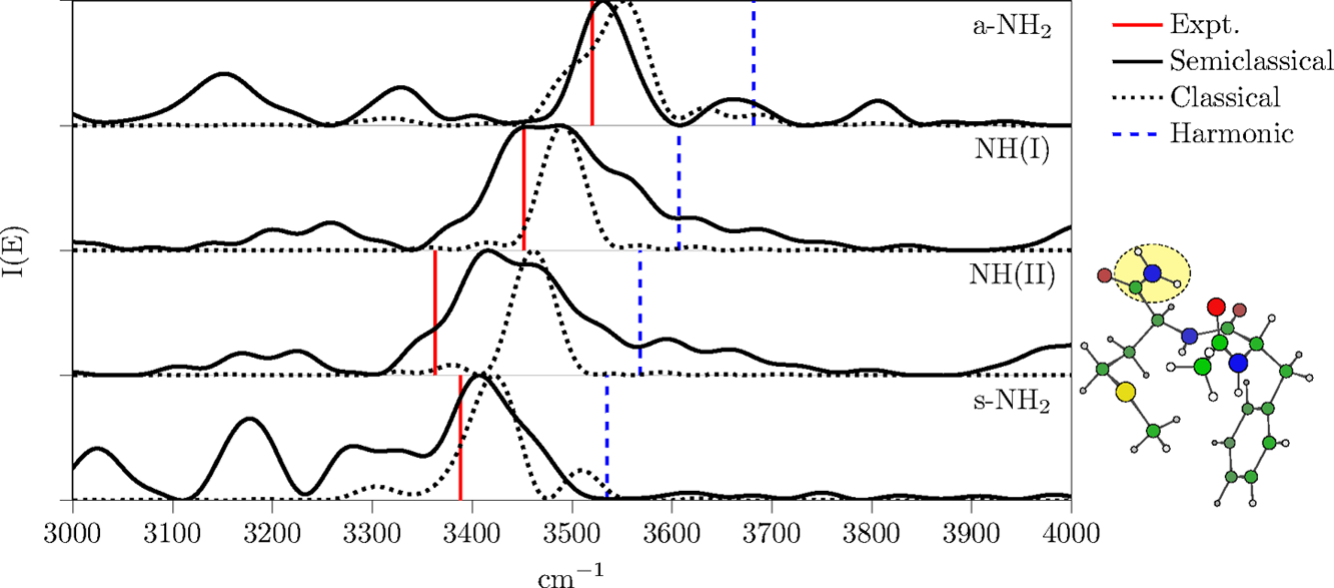
Ac-Phe-Met-NH_2_ amide group related vibrational stretching power spectra. Vertical continuous sticks are the experimental values,^114^ while vertical dashed sticks are the harmonic approximation frequencies. Continuous lines are for MC-DC SCIVR simulation using the Hessian NGas approximation and dotted lines are for the quasi-classical simulation on the same *ab initio* potential (see main text).

Figure 3 shows the power spectra of the selected amide group stretching modes using different Hessian approximations. On each panel is reported the signal of each mode after a suitable combination of coherent states.^96^ Continuous lines are for MC-DC SCIVR simulations where Hessians have been calculated at each time-step and are labeled as “all Hessians.” Dashed lines are for our NGas approximation presented above, that is, with the inclusion of the gradients in the set of neuron variables. The dotted line is the so-called “SH” approximation,^115^ where the Hessian is constant and it is equal to the equilibrium geometry one. The NGas simulation is very similar to the exact, especially for the higher-dimensional subspace. However, the SH approximation is quite good if one takes into account how drastic the approximation is. Nevertheless, the main problem of the SH approximation is that for the higher-dimensional subspace containing the NH(I) and NH(II) stretches, it does not allow for a definitive assignment, while in the case of the NGas spectra, a main peak is present, despite the numerous overtone side peaks. We can confirm that these side peaks of smaller intensities are of the type of overtones or combination bands by comparing the NGas spectra with the classical ones in Figure 4.

Quasi-classical spectra are obtained by Fourier transforming the velocity–velocity correlation function of a constant energy trajectory (*NVE*), which is the same one employed for the MC-DC SCIVR calculations, that is, trajectories starting from the equilibrium geometry and with kinetic energy equals to the harmonic zero-point energy (ZPE). While these types of classical simulations provide frequency values with anharmonic corrections because the *ab initio* trajectory accounts for the shape of the PES, these values are restricted only to the fundamental transition frequencies and higher harmonics. Instead, semiclassical simulations, such as MC-DC SCIVR, provide the full collection of eigenvalues as in eq 14, and all type of transition frequencies can be obtained by the difference between the eigenvalues. Thus, the semiclassical power spectrum includes not only the fundamental frequencies but also anharmonic overtones, combination bands, and the ZPE value on an absolute scale. For these reasons, the MC-DC SCIVR spectra of Figure 4 (continuous lines) present several more spectroscopic features than the classical ones (dashed lines). However, it is still possible to compare the two of them with the experiments on the fundamental frequency values. The comparison is reported in Table 4 and Figure 4, where the experimental values^114^ are reported as a red continuous stick spectrum, while the harmonic estimates are the dashed blue sticks. Overall, we can observe in Figure 4 that the semiclassical simulations present broader peaks than the classical ones. The classical peak width is what is expected from the Fourier transform of a ∼0.73 ps simulation. We do not pursue longer trajectories because the quantum accuracy of the semiclassical approximation would deteriorate for longer simulations. We also decided to not apply any artificial exponential constant decay (Gaussian filter) to avoid any sort of biasing. In Figure 4, the semiclassical signals are broader in the case of the NH(I) and NH(II) stretches, as expected, because of the numerous strongly coupled floppy modes. Specifically, the more intense peaks in the NH(I) and NH(II) panels represent the convolution of a series of overtones coupled to the numerous floppy modes, while the other side peaks, which are absent in the classical spectrum, are a combination or overtone bands of other modes. In fact, both the subspace subdivision and the filtering process provided by the combination of coherent states^96^ can only partially filter the numerous eigenvalues which are present in a given energy window of a 132-dimensional power spectrum. Clearly, in Figure 4, these side peaks are less intense at higher frequencies because the trajectory energy shell is at the level of the harmonic ZPE value, where the Fourier transformed coherent state is centered.

**Table 4:** Selected Amide Group Vibrational Stretching Fundamentals of the Ac-Phe-Met-NH_2_ Peptide at Different Levels of Approximation^a^

modes	all Hessians	NGas	DBH^16^	classical	harmonic	exp^114^
aNH_2_	3548	3530	3490	3552	3682	3520
NH(I)	3448	3456	3480	3490	3607	3452
NH(II)	3412	3416	3300	3461	3568	3363
sNH_2_	3426	3406	3360	3422	3535	3388
MAE	29	21	37	51	167	0.0

a The first column reports the type of stretch, the second reports the MC-DC SCIVR frequencies without any Hessian approximation, the third and the fourth columns, respectively, report the NGas and the DBH approximated Hessians semiclassical frequency values, the fifth column is the quasi-classical frequencies of vibration, the sixth column is the harmonic results, and the last column shows the experimental values.^114^ In the last row, the MAE with respect to the experimental values is reported for each method.

Table 4 summarizes the results in Figure 4 with the additional results of the semiclassical MC-DC SCIVR simulation obtained using the Hessian database approximation.^16^

The comparison between different levels of calculation shows that classical and semiclassical results are systematically more accurate than the harmonic ones, while the semiclassical ones are further more accurate with respect to the classical ones. The semiclassical reference is reported in the second column of Table 4, where the calculations have been performed without any Hessian approximation but using directly the *ab initio* values. The third and fourth columns report, respectively, the NGas and the DBH approximated Hessian semiclassical values. For the NGas simulation, 200 neurons and 200 *ab initio* Hessians have been employed, while the DBH results are obtained with 300 *ab initio* Hessians.^16^ At this level of comparison, we think it is not possible to assert which of the Hessian approximations, either the NGas or the DBH one, is more appropriate for spectroscopic analyses with the MC-DC SCIVR method. Actually, the NGas MAE with respect to the experimental values in Table 4 is slightly smaller than calculating all *ab initio* Hessians. This is clearly due to a compensation of effects, which include the level of *ab initio* theory. Eventually, given the NGas and DBH MAE of Table 4, both of them are accurate enough for semiclassical calculations, considering that any semiclassical simulation strongly depends on the level of *ab initio* theory and that the Fourier transform broadening is about ∼20 cm^–1^ for a typical semiclassical trajectory simulation, where the total time is on the order of picoseconds.

## Conclusions

Given the importance of an accurate method for approximating instead of calculating the Hessian matrix during MD simulations, we have investigated the possibility to employ a slightly customized NGas algorithm that allows us to compute the Hessian matrix of the potential energy along a MD simulation. After presenting the method, we have tested its accuracy compared to other algorithms already present in the literature.^14−16^ Then, we applied it to speeding up the calculation of semiclassical spectra, where the Hessian calculation is mandatory at each MD time-step. We find that the NGas algorithm can be ∼20% more accurate than other methods for simulations of molecular systems whose trajectories overlap and cross significantly. Furthermore, it appears that the NGas method may require far fewer *ab initio* Hessian calculations to provide the same accuracy as competitive methods. However, some caveats must be taken into account. First of all, if one aims to study a single short trajectory of a large molecular system (such as Ac-Phe-Met-NH_2_), it appears that the NGas method is just as accurate as the DBH approach that our group presented recently.^16^ As a matter of fact, in such cases, the NGas method provides a solution that is similar to that proposed by DBH. Furthermore, if the user can afford to compute only very few Hessian calculations along a nonoverlapping trajectory, it is recommended to add gradients of the potential to the NGas training set. This set up is slightly more robust than the DBH method with respect to the number of *ab initio* Hessians. Second, while at high dimensions, all methods scale favorably, the NGas method would suffer from longer simulations and higher number of neurons. However, we expect that this feature should still compensate for the time spent for the *ab initio* calculation of all Hessians. We did not pursue the simulations of molecular systems significantly larger than Ac-Phe-Met-NH_2_ because the Hessian calculations at each time-step would be out of reach for standard computational power. The third caveat is that the DBH method can also be performed on-the-fly, while the NGas one is necessarily a postprocessing method. This means that in the DBH method, the number of *ab initio* Hessian calculations can be automatically determined during the dynamics if one applies the method to the available database at each time-step and increment the database during the dynamics, while in the case of the NGas method, it has to be fixed a priori. The last caveat is that the DBH parameter ρ is system-dependent. Also, ρ ensures that the approximated Hessians are close enough to the trajectory, but it does not allow to control the number of Hessians to compute. On the other hand, the NGas method requires as input the number of Hessians one is willing to compute, but it does not assure that the neuron locations would be close enough to the trajectory. Nevertheless, both these shortcomings can be mended by a preliminary trial and error calculation. Eventually, for semiclassical spectroscopic calculations, we conclude that both methods are accurate. We also tested the SH approximation and confirm that this choice should be avoided or employed as a preliminary calculation together with a classical power spectrum calculation. Finally, in this work, we have also shown that our DC SCIVR technique implemented by reasonable approximations can allow for power spectrum calculations with the inclusion of quantum nuclear features of systems as large as small peptides. As a future perspective, our NGas method could be interfaced with methods that generate a local fit of the potential, such as the GPR-PES method.^22^ In fact, the trajectory geometries within a Voronoi cell can be used to train a GPR model and better approximate the Hessian matrix within the same cell. This approach would allow for more reliable Hessian estimates within the current limitations of applications of the GPR-PES methodology.

## Code Availability

The codes developed for this work are freely available on github at: https://github.com/ganmichele/hessapprox.

## Supporting Information Available

Pseudocodes for NGas, DBH, and CFD-Bofill methods, scatter plots of neurons and trajectory geometries for H_2_O and HCOH molecules, and a plot of the Hessian matrix element absolute error *versus* the trajectory step(PDF)
NGas intermediate steps on a 2D plane (MP4)
