# Supplementary material for: Unsupervised Machine Learning Neural Gas Algorithm for Accurate Evaluations of the Hessian Matrix in Molecular Dynamics
Source: J Chem Theory Comput. 2021 Oct 27;17(11):6733–46. doi: 10.1021/acs.jctc.1c00707 (PMC8582248; doi:10.1021/acs.jctc.1c00707)
Supplement: Supplementary file 1 — ct1c00707_si_001.pdf [file ct1c00707_si_001.pdf]

Supplementary Material for: An Unsupervised Machine Learning Neural Gas Algorithm for Hessian Matrix Evaluations in Molecular Dynamics

Michele Gandolfi and Michele Ceotto*

Dipartimento di Chimica, Università degli Studi di Milano, via Golgi 19, 20133 Milano, Italy

E-mail: michele.ceotto@unimi.it

Hessian approximation codes

The codes for the Hessian approximation methods employed in this work are freely available on github at https://github.com/ganmichele/hessapprox. We also report here the barebone pseudocodes.

NGas pseudocode

This code generates a database of configurations (called *qtilde*) for which the Hessian matrix calculation is requested. Also, the program provides a list of indexes (called *nearest*) which assign to each neuron each configuration in the input trajectory.

Listing 1: NGas

```
SET tau=0, epochs             # current epoch number, number of epochs
```

```
SET amin, amax, lmin, lmax  # neural gas parameters. Recommanded values: 0.3, 0.05, 30, 0.01
SET trajsteps, df           # number of data points, dimension of the gas space
SET numneur                 # number of neurons
SET numneighbors            # numneighbors*lambda = number neurons to update. Recommended 10

READ q(:trajsteps,:df)      # read array of trajectory coordinates

SET M = MAX( q)             # 1-D array of maximum values in the columns of traj
SET m = MIN( q)             # 1-D array of minimum values in the columns of traj
SET Q = (q - m) / (M - m)

CALL even_sample( Q, Qtilde) # evenly sample neurons "Qtilde" from "Q"

DO epochs iteration
  # update the parameters alpha and lambda
  SET alpha  = amin * (amax / amin)^(tau/epochs)
  SET lambda = lmin * (lmax / lmin)^(tau/epochs)

  CALL shuffle( Q) # randomly shuffle the scaled trajectory

  # decide how many neighbors "nh" are updated (speeds up optimization)
  IF ( ceiling(lambda)*numneighbors > numneur )
      SET nh=numneur
  ELSE
      SET nh=ceiling(lambda)*numneighbors
  END IF

  DO iterate over the trajectory points index "i"
    CALL distance( Q(i,:), Qtilde, dist) # get array of distances from the neurons
    CALL sort( dist, Ki)                 # get neuron order vector, closest to furthest wrt Q(i,:)
    DO iterate over the near neurons "nh" with index "j"
      SET Kij = Ki(j)
      SET Qtilde(Kij,:) = Qtilde(Kij,:) + alpha*exp( -j/lambda) * (Q(i,:) - Qtilde(Kij,:))
    END DO
  END DO

  SET tau = tau + 1
END DO # epochs loop

# recenter neurons to the center of mass
CALL distance( Qtilde, Q, distmat)     # computes the distance matrix neurons vs trajectory
```

```
CALL argmin( distmat, nearest)          # get array of indexes of closest steps from the neurons
SET Qtilde(j,:) = MEAN( Q( nearest,:))  # vector of means to get "center of mass"

SET qtilde = Qtilde * (M - m) + m

SAVE qtilde    # write optimized neurons
SAVE nearest   # write which trajectory points belong to which neuron
```

DBH pseudocode

This code generates a database of configurations (namely *DBq*) for which one need to compute the Hessian matrix. Also, the program provides a list of indexes (namely *nearest*) which assign each configuration in the input trajectory to a configuration in the database.

Listing 2: DBH

```
SET rho                  # choose database parameter
SET steps, df            # number of trajectory steps, degrees of freedom

READ q(:steps,:df)         # read array of trajectory coordinates

# initialize empty database of coordinates; DBq may have fixed or variable size
SET DBq(:,:df)     = empty

SET nearest(:steps) = empty
SET onthefly     = FALSE

# search the database for a match
DO iterate over the trajectory points with index "i"
      SET add2db = TRUE
      DO iterate over the database points with index "j"
    SET dist = MAX( q(i,:) - DBq(j,:))
      IF ( dist < rho )
              add2db = FALSE
              BREAK INNERMOST LOOP
      END IF

  END DO  # end of datasase search

```

```
      IF ( add2db == TRUE )
        # determine closest DBq to q
      distmin = infinity
      DO iterate over database points with index "j"
        # compute distance between q and DBq with a distance function
        dist = DISTANCE( q(i,:), DBq(j,:) )
      IF ( dist < distmin )
              distmin = dist
                nearest(i) = j # DBq(j) is nearest to q(i)
      END IF
      END DO
  ELSE
    APPEND q(i,:) to DBq
    SET nearest(i) = j+1
  END IF

END DO # end of trajectory loop

# if you do not need the hessians "on the fly", you should
# locate the "nearest" DBq on the whole trajectory
IF ( onthefly == FALSE )
      CALL distance( DBq, q, distmat)  # computes the distance matrix DBq vs trajectory
      CALL argmin( distmat, nearest)   # update nearest variable with closest DBq from traj
END IF

SAVE DBq       # write optimized database
SAVE nearest # write which trajectory geometry belogs to which entry in DBq
```

CFD-Bofill pseudocode

Assuming that Hessian matrices are calculated every N steps of the trajectory, this pseudocode extrapolates the Hessians up to the next exact Hessian evaluation, and provide a full list of Hessian for each time-step.

Listing 3: Bofill

```
SET lambda          # CFD parameter
SET steps, df       # number of trajectory steps, degrees of freedom
SET N               # number of Hessians to predict for each one given
```

```
READ q(steps,df)     # read array of trajectory coordinates
READ g(steps,df)     # read array of energy gradients along trajectory q
READ H(steps,df^2)   # read array of hessians, empty except once every N steps

DO iterate over the trajectory points with index "i"

  IF ( H(i,:) is not empty )
      GO TO next iteration
  ENDIF

  SET dq = q(i,:) - q(i-1,:)
  SET dg = g(i,:) - g(i-1,:)
      SET h  = H(i-1,:)
      SET R = 2 * ( dg - dot(h, dq))

  IF ( lambda is not set )
            # if lambda not given, use default Bofill value
      SET lam = 1.0 - ( dot(R,dq)^2 / ( dot(R,R) * dot(dq,dq) ))
  ELSE
      SET lam = lambda
  END IF

  SET h      = h + (1-lam) * outer(R,R) / dot(R,dq)  # outer is outer product
  SET H(i,:) = h - lam * dot(R,dq) / dot(dq,dq)^2 * outer(dq,dq)

END DO
```

Hessian approximation at each time step

H_2O

We show here some more results of the Hessian approximations for the simulation of water molecule in the gas phase. We fix 150 neurons for a 3000 steps trajectory and optimize the NGas position with 30 epochs. The neurons are initialized as trajectory points by selecting one neuron every 20 time-steps. In Fig. (1) we show the optimal positions of the neurons on a short portion of the trajectory. To obtain Fig. (1) we ran the simulation of H_2O and

discard the normal mode associated with symmetric stretching. Then we optimized neurons locations on the $(q_1(t), q_2(t))$ sub-dimensional trajectory. In this way it is easy to see how the neurons are located out of the trajectory when the latter is curved, while the neurons are along the trajectory when it is linear. In Fig. (2) we report the error on each Hessian matrix along the trajectory using the three approximation schemes NGas, DBH and Bofill.

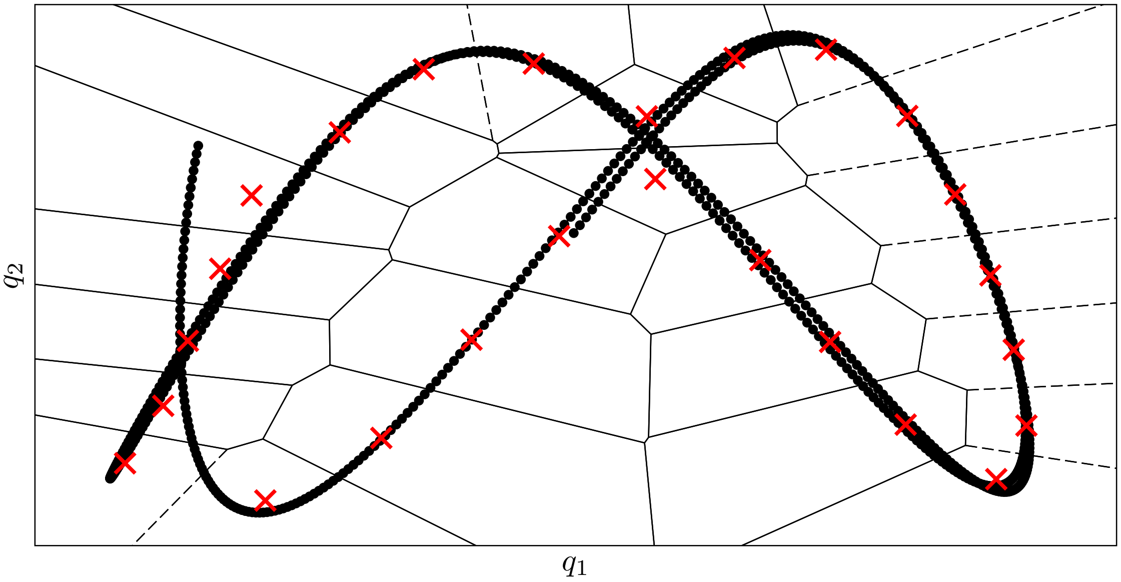

Figure 1: H_2O classical trajectory projection. The black dots are the trajectory positions for the first two normal modes (q_1, q_2). The red crosses are the neuron locations. The (q_1,q_2) plane is divided into Voronoi cells according to the neurons locations. As some cells are infinite in size, their infinite boundaries are drawn with dashed lines.

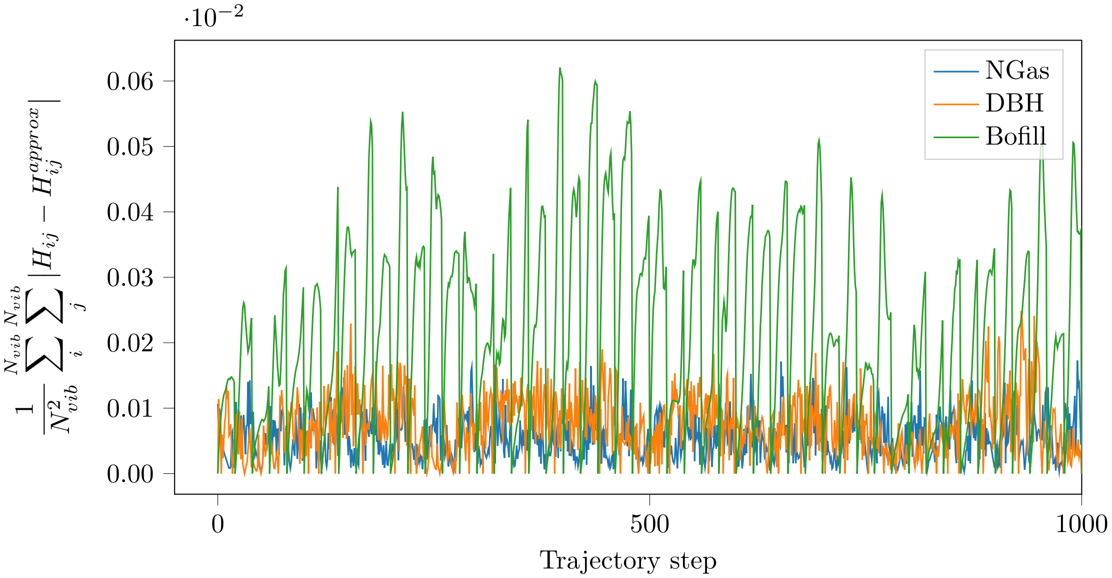

Figure 2: Plot of the deviation of the Hessian matrix elements along the first 1000 time-steps of the water molecule simulation. The Bofill method is reported in green, DBH in orange and the NGas in blue

HCOH

Here we report some more details about our formaldehyde molecule simulation. We fix 150 neurons for a 3000 steps trajectory and optimize the Neural Gas position with 30 epoch cycles. The neurons are initialized on top of some trajectory points by selecting one neuron every 20 time-steps. The final positions of the neurons are reported in Fig. (3) for a bi-dimensional cut of the 6 dimensional space. Differently form Fig. (1), here the neurons locations have been optimized on the full dimensional system (6 vibrational degrees of freedom).

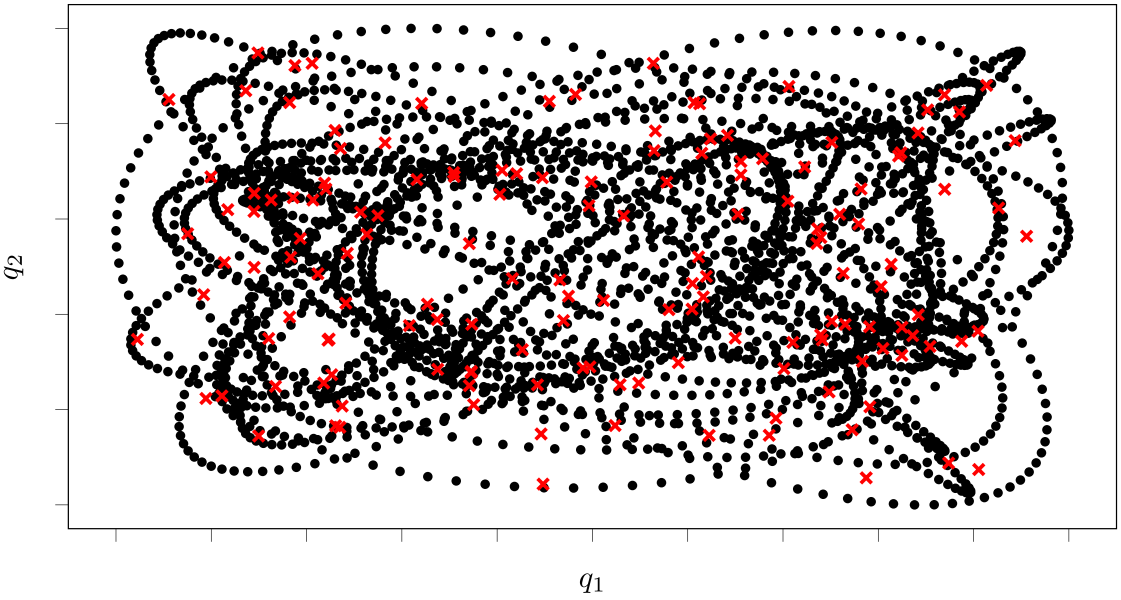

Figure 3: HCOH classical trajectory bi-dimensional projection. The black dots are the trajectory positions for the first two normal modes (q_1, q_2). The red crosses are the neuron locations.

Fig. (4) reports the Hessian matrix elements deviation from the exact ones for the different Hessian approximation methods.

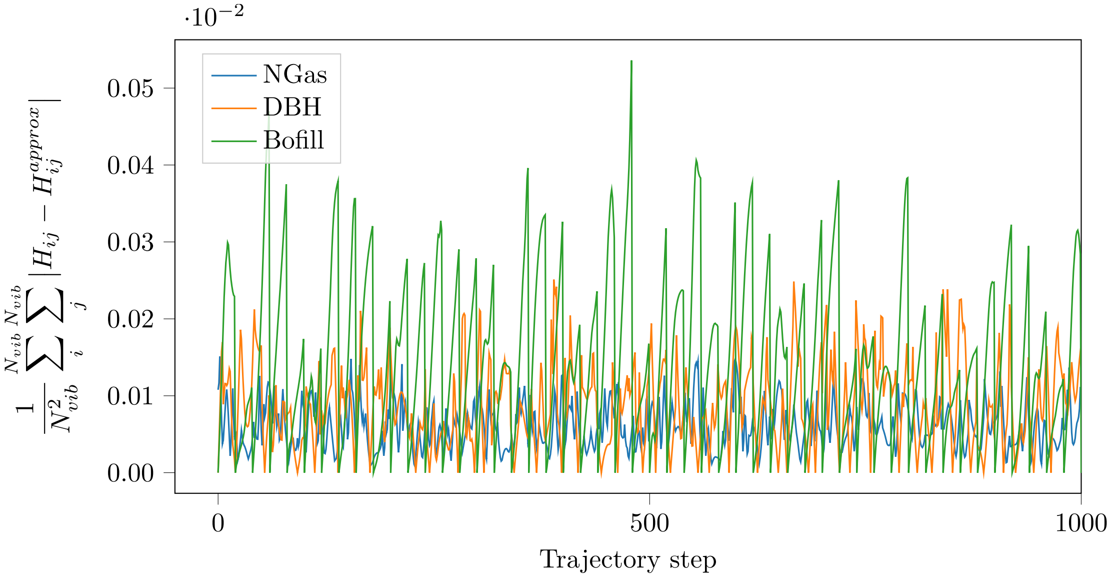

Figure 4: The same as in Fig. (2) but for formaldehyde.
